# Factors Driving Amyloid Beta Fibril Recognition by Cell Surface Receptors: A Computational Study

**DOI:** 10.3390/molecules30204116

**Published:** 2025-10-17

**Authors:** Olivia Slater, Maria Kontoyianni

**Affiliations:** Department of Pharmaceutical Sciences, Southern Illinois University, Edwardsville, IL 62026, USA; oslater@siue.edu

**Keywords:** Alzheimer’s disease, toll-like receptors, beta-amyloid, *A*β fibrils, Receptor for Advanced Glycation End products (RAGE)

## Abstract

Alzheimer’s disease (AD) has been studied extensively and is characterized by plaques deposited throughout the brain. Plaques are made of beta-amyloid (*A*β) peptides which have undergone fibrillogenesis to form insoluble *A*β fibrils (*fA*β) that are neurotoxic. Receptor for Advanced Glycation End end products (RAGE), toll-like receptors (TLRs) 2 and 4, and co-receptor CD14 recognize negatively charged binding regions on *fA*β to activate microglia and release proinflammatory cytokines. In this study, we used two experimentally resolved *fA*β structures (type I and II) isolated from AD brain tissue to elucidate binding patterns of *fA*β with RAGE, TLR2, TLR4, and CD14 and investigated whether binding was affected by fibril structure or system pH. Receptors TLR2 and RAGE formed tight complexes with both type I and II fibrils, while TLR4 showed selectivity for type I. CD14 binding was less tight and selective for type II. Binding was pH dependent for CD14, TLR4, and RAGE but not TLR2. We explored the effects of familial mutations on fibril structure to determine whether mutants of type I or II structures are feasible. Finally, we investigated whether mutations affected binding interactions of *fA*β with proteins. The Arctic (Glu22Gly), Dutch (Glu22Gln), and Iowa (Asp23Asn) mutations showed similar effects on binding affinity. Italian (Glu22Lys) mutations abrogated binding, whereas type I and II fibrils with Flemish (Ala21Gly) mutations were not shown to be feasible. Results highlight the adaptability of immune receptors in recognizing damaging molecules, with fibril structure and pH being the main recognition determinants predicated on disease progression. In silico mutations showed that aggregates similar to type I and II structures were plausible for some familial mutations.

## 1. Introduction

Alzheimer’s disease (AD) is a neurodegenerative disorder that plagues more than 57 million people and is projected to increase to 153 million by the year 2050 [1]. Disease hallmarks include neurofibrillary tangles and plaques that are deposited throughout the brain, while lowered brain pH has also been correlated with age and AD progression [2]. Plaques are made up of beta-amyloid (*A*β) peptides; increased plaque load leads to increased cognitive decline and memory loss. *A*β is produced by proteolytic cleavage of the amyloid precursor protein. Samples from AD patients showed *A*β peptides with between 37 and 43 amino acids, with the most abundant forms being full-length *A*β_40_ and *A*β_42_ [3,4]. *A*β_42_ is less soluble and more prone to aggregation, forming neurotoxic oligomers, protofibrils, and fibrils, which are precursors to amyloid plaques. *A*β_42_ filaments are most common in individuals with sporadic or familial AD [5].

There are several familial mutations associated with AD that can affect peptide aggregation and the rate at which fibrils are formed. For example, Arctic (Glu22Gly), Dutch (Glu22Gln), and Iowa (Asp23Asn) mutations promote fibril nucleation of *A*β_42,_ whereas Flemish (Ala21Gly) or Italian (Glu22Lys) mutations do not [6]. The Flemish mutation showed reduced fibrillogenesis in vitro versus the Arctic, Dutch, Italian, and Iowa mutations, which have been shown to enhance fibrillogenesis [7,8]. However, the effects of mutations on fibrillar structure is not clear. Further, how these mutations affect binding interactions with proteins has not been thoroughly investigated.

Receptors on the surface of immune cells bind *A*β fibrils (*fA*β), and the effects can be either protective or damaging. Direct interactions between *fA*β and CD14, TLR2, and TLR4 were demonstrated through cellular adhesion assays [9,10]. Further evidence of *fA*β–CD14 was observed using flow cytometry and confocal microscopy [11]. Direct integration with TLR2 was demonstrated with real-time Surface Plasmon Resonance (SPR) spectroscopy and pull-down assays [12]. SPR and competition assays using mAb B10 proved *fA*β–RAGE interactions occur through the extracellular VC1 domain [13]. Fibril recognition by toll-like receptors (TLRs) 2 and 4 and co-receptor CD14 stimulated phagocytosis in microglia, which is neuroprotective [9,14]. In early stages of the disease state, *A*β interactions with Receptor for Advanced Glycation End products (RAGE) slow nucleation and the formation of filaments that form plaques. However, *fA*β interactions with TLR2, TLR4, and RAGE activate microglia to release proinflammatory cytokines that inhibit phagocytosis and fibril degradation, and promote neuroinflammation and toxicity [15,16]. The underlying mechanisms of neuroprotection and degeneration are not fully understood, and there are contrasting results on the effects of fibril interactions with immune receptors, but it is not clear why [17,18]. Receptor function has been postulated, but the possibility of receptors exhibiting selectivity for certain fibril structures has not been explored. Further, acidic extracellular environments promote plaque formation and impair inflammatory responses in microglia. Acidic pH in the endocytic and lysosomal pathway promotes fibril formation [19]. *A*β accumulation in lysosomes results in microglial cell death and ultimately in plaque formation [20,21]. Reduced pH during inflammation accelerates fibril formation as the pH approaches the isoelectric point of *A*β. It has been shown that the primary nucleation has a strong pH dependence, whereas secondary nucleation and elongation rate constants are not pH dependent [22]. Increased pH during fibril formation of α-synuclein has been confirmed experimentally. Thus, mild acidosis observed in AD brains could affect interactions with immune receptors on the microglial surface. Nevertheless, how these pH shifts impact fibril structure and surface properties and by extension receptor interactions is not understood, but these shifts remain significant in cellular responses such as synaptic dysfunction and neurotoxicity. Thus, mild acidosis observed in AD brains could affect interactions with immune receptors on the microglial surface [2].

Studies that used monoclonal antibody (mAb) B10 confirmed that electrostatic interactions between cationic residues of the antigen binding site and anions within *fA*β’s binding epitope were responsible for affinity [13]. RAGE binds *fA*β similar to B10 through the extracellular VC1 domains [13]. Likely, *fA*β would form similar interactions to those observed within the RAGE–DNA complex. Specifically, lysines 37, 39, 43, 107, and 123, arginines 29 and 218, and Tyr118 hydrogen bonded with the phosphate backbone of purine or pyrimidine bases non-selectively [23]. Amino acid interactions have not been reported for *fA*β binding with TLR2, TLR4, or CD14. However, these receptors recognize damage associated molecular patterns. Therefore, interactions are likely to occur between positively charged residues of the receptors and the anionic binding site of *fA*β.

Recently, *fA*β structures were isolated from AD brain tissue and resolved using cryo-electron microscopy (cryo-EM) (PDB IDs: 7Q4B and 7Q4M) [5]. Both fibrils consisted of 10 full-length *A*β_42_ peptides that were divided evenly between two s-shaped protofilaments, but fibril morphologies differed. Type I exhibited pseudo-2_1_ symmetry and was left-hand twisted. The s-shaped domains included residues Phe19–Ala42 and the first eight residues were disordered. Hydrogen bonds were formed along the fibril stack between residues Glu11 and His13, and each peptide had five β-strands. Type II exhibited C2 symmetry and was left-hand twisted. The s-shaped domains included residues Phe20–Ala42 and residues Asp1–Glu11 were disordered. In both PDBs, the disordered regions were not included. Salt bridges were formed between Lys28 side chains and carboxy termini, and each peptide had four β-strands. Both fibrils had acidic residues Glu22 and Asp23 of the binding epitope exposed to solvent.

In this work, we addressed four questions: (**i**) What are the differences structurally and energetically between type I and II *fA*β? (**ii**) Is *fA*β recognition by cell surface receptors affected by fibril structure or system pH? (**iii**) Are mutants of type I or II structures feasible and what are the effects of familial mutations on *fA*β structures? (**iv**) Could mutations affect binding interactions with proteins? Toward question one, we generated low-energy conformations of type I and II fibrils using Molecular Dynamics (MD) simulations and performed docking experiments of *fA*β with CD14, TLR2, TLR4, and RAGE in order to elucidate binding patterns. The reasons for selecting CD14, TLR2, TLR4, and RAGE include the following: (1) pathophysiological relevance of *fA*β–receptor complexation as outlined above; (2) primary receptors for signal transduction and/or phagocytosis of *fA*β [11,12]; (3) availability of resolved experimental structures; and (4) limited flexibility of the extracellular domain(s) that bind *fA*β [24,25,26,27]. To address the second question, we performed pH-dependent computational alanine scanning to identify interaction hot spots of type I versus II complexes and extracted differences in free energies of binding (ΔΔG_bind_) to estimate binding affinities at pH 5 and 7. Toward three, we assessed the feasibility of mutant structures having the same morphology as type I or II fibrils by generating Ala21Gly, Glu22Gly, Glu22Gln, Glu22Lys, and Asp23Asn mutants of type I and II *fA*β in silico, and performed MD simulations for comparison of energetics and non-bonded interactions with wild-type structures. Toward four, we performed combinatorial amino acid scanning mutagenesis on *fA*β residues within bound complexes, followed by MM/GBSA calculations to quantify energetic effects of mutations on binding affinity.

## 2. Results and Discussion

### 2.1. Type I and II FAβ Structures

#### 2.1.1. What Are the Differences Structurally and Energetically Between Type I and II *FA*β?

To observe how the fibril structures behaved under normal conditions, we performed five 200 ns MD simulations for type I and II structures (10 total). We selected simulation parameters guided by studies focused on fibril dynamics [28,29]. Both fibrils had lower potential energies from 0 ns to 200 ns, and there was no change in potential energy when simulations were extended an additional 300 ns, which indicated conformations remained stable. We visually assessed fibril conformations using simulation frames taken every ns; because no differences between runs were observed, we selected representative snapshots for discussion. We further evaluated root mean squared deviation (RMSD), root mean squared fluctuation (RMSF), radii of gyration (R_g_), and solvent accessible surface area (SASA). Briefly, the average compactness of the conformation is represented by R_g_ as the average squared distance of heavy atoms from the fibril’s center of mass. Changes in R_g_ indicate conformational shifts. We assessed flexibility of the s-shaped domains compared with the disordered regions using RMSF which measures the displacement of backbone atoms from the initial cartesian coordinates at time zero on a per-residue basis. We also compared our results with experimental structures to identify which secondary elements, structural motifs, and non-bonded interactions were maintained, lost, or gained and used surface maps to assess charge distributions at the binding motifs.

The potential energies of type I and II fibrils were approximately −9.4 × 10^5^ kJ/mol and −1.4 × 10^6^ kJ/mol, respectively Both fibrils had average C-alpha RMSD of 0.2 nm, which indicated backbone atoms did not deviate significantly from the initial coordinates (Figure S1a). RMSF curves showed rigidity within the s-shaped domains compared with amino acids at the termini, which was expected. Specifically, the type I curve had peaks between 0.40 nm and 0.74 nm that corresponded to Gly9–Val12, and the type II curve had peaks between 0.42 nm and 1.20 nm that corresponded to residues Val12–His14 (Figure S1b). R_g_ curves were linear, indicating structures did not undergo conformational changes (Figure S1c). Fibrils had similar solvent accessibility with average SASA equal to 139.9 nm^2^ for type I and 138.5 nm^2^ for type II *fA*β structures.

**Figure 1:**
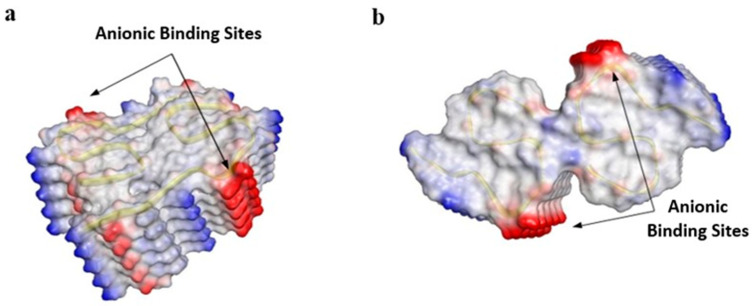
Surface charge maps of (**a**) type I and (**b**) type II fibrils. Red indicates negative charge, blue indicates positive charge, and white represents neutral amino acid residues. Fibrils are composed of two s-shaped protofilaments that contain a binding epitope that includes 20 carboxylic acids from Glu22 and Asp23 residues stacked along the fibril axis.

Secondary structures were maintained and included 5 and 4 β-strands per peptide for types I and II, respectively. Residues within anionic binding sites had the same interactions as reported for the experimental structures [5]. We refer to interactions between residues within the same protofilament as ‘intrasubunit’ interactions, and interactions between residues on opposite protofilaments ‘intersubunit’ interactions. For type I, inter- and intrasubunit interactions were formed between Glu11 and His13. For type II, salt bridges were formed between NH_3_^+^ of Lys28 and the C-termini which stabilized the interface between protofilaments. Residues 21–23 formed the same non-bonded interactions for type I and II structures. Specifically, main chain NHs of Ala21 residues hydrogen bonded with carbonyl oxygens of Phe20 residues, atoms of Glu22 were not involved in non-bonded interactions with fibril residues, and hydrogen bonds formed between main chain NHs of Asp23 and carbonyl oxygens of Val24 residues. The 20 anions of the binding epitope (10 Glu22 plus 10 Asp23 residues) were exposed to solvent and created strong negative surface charge patches along the fibril axes (Figure 1a,b).

**Figure 2:**
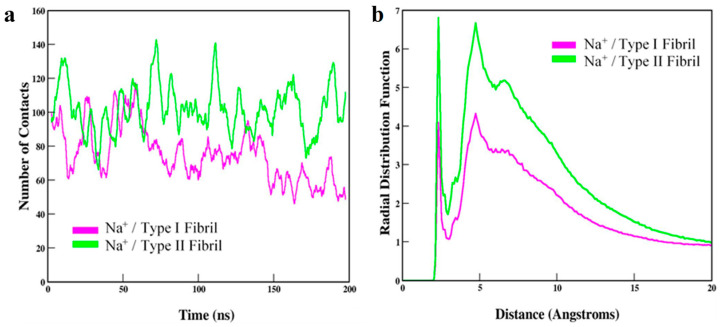
(**a**) The number of contacts made between Glu22 or Asp23 side chains of binding epitopes and Na^+^ ions of the solvent. Contacts are shown as averages calculated every 10 ns. (**b**) Radial distributions of Na^+^ ions as a function of the distance from residues of the binding epitope for type I and II *fA*β structures.

Calcium promotes aggregation of *A*β and fibrillation of *A*β_42_. Magnesium could play a neuroprotective role by modulating *A*β clearance. Sodium impacts the rate and morphology of aggregation and plausibly exacerbates AD pathology through oligomer production. In this study we elected to focus on sodium. Throughout simulations, type II fibril formed more contacts between Glu22 and Asp23 with Na^+^ ions compared with type I fibril (Figure 2a). Radial profiles of type I and II structures had two distinct peaks at 2.5 Å and 5 Å. Both curves steadily decreased toward g(r) 1.0 at 20 Å, which indicated that the systems were at equilibrium. Type I structure had lower peak heights at 4.1 and 4.3 versus type II with peaks reaching approximately 6.8. These results indicated ordered Na^+^ molecules at the binding epitope and that type I interacted less with solvent ions compared with type II (Figure 2b). Alternatively put, these results could be interpreted as coordination of metal ions through non-bonded networks of COO^−^ groups of Glu22 and Asp23 side chains, and coordination is stronger for type II compared with type I *fA*β. Our study was limited to monovalent Na^+^, but in vivo divalent cations play critical roles in brain function also. Further, these results suggest that the binding epitopes of type I and II fibrils have slightly different electrostatic character, which could potentially affect long-range interactions that are important for binding proteins.

In summary, type I and II configurations had the same structural dynamics and maintained stable conformations, but type II fibril was lower in energy despite having fewer ordered residues because of the stabilizing non-bonded interactions between protofilaments at the interface. Structures maintained secondary elements and intramolecular interactions reported from crystallography experiments. The binding epitopes were exposed to solvent, and type II anions were more strongly coordinated with Na^+^ ions. Importantly, these results suggest differences in electrostatics of the anionic binding epitopes between type I and II structures.

#### 2.1.2. Is *FA*β Recognition by Cell Surface Receptors Selective Dependent upon Fibril Structure or System pH?

In order to address whether fibril structure affects protein binding, we docked low-energy conformations of type I and type II *fA*β into CD14, TLR2, TLR4, and RAGE homodimer. Interactions at the *fA*β–mAb B10 interface have been confirmed experimentally [13] and were used as validation of our methodology. To identify interfacial residues that contribute to binding affinity and pH selectivity, we performed in silico alanine scanning of interfacial residues and extracted changes in free energies of binding as a function of pH (ΔΔG_bind_) and in response to mutations (ΔΔG_mut_). Residues with ΔΔG_mut_ >1 kcal/mol were considered stabilizing, and those with ΔΔG_mut_ ≥ 2 kcal/mol were considered interaction hot spots [30]. Because electrostatics are highly important for fibril–protein interactions, calculations were performed across a pH window of 2 to 12 in order to address potential effects of pH on binding affinity. We considered disease relevant pH range 5 to 7 in our analyses [2]. There are several binding modes that are feasible for the complexes under investigation. We selected representative binding modes using visual inspection and alanine scanning to identify complexes where the antigen’s binding eptiope was present at the interface and formed stabilizing interactions.

**Figure 3:**
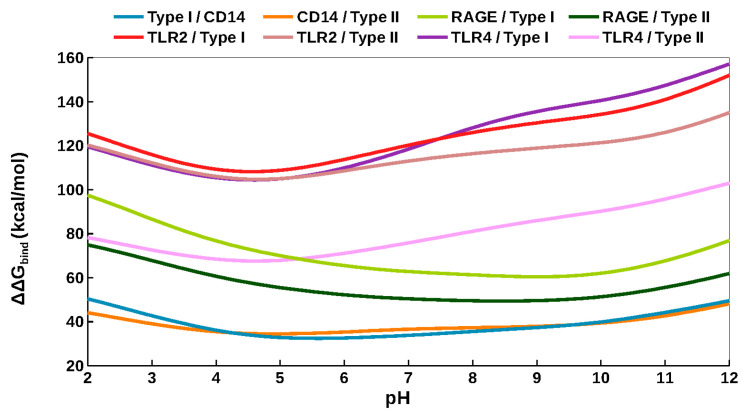
Changes in binding free energies of *fA*β–receptor complexes as a function of pH.

Figure 3 shows the changes in free energy of binding as a function of pH. The binding curve for type I *fA*β–CD14 was flat, and there were no hot spots at either pH 5 or 7 (Figure 3 and Figure S2a). The type II *fA*β–CD14 curve showed ΔΔG_bind_ increased approximately 3 kcal/mol as pH increased from 5 to 7 (Figure 3). There were two hot spot residues at pH 7, including Arg92 and Lys173, which contributed 2.8 and 2.4 kcal/mol, respectively. Both residues formed electrostatic interactions with Asp23 residues of *fA*β that were weakly stabilizing at lower pH (Figure S2b).

Curves representing *fA*β–RAGE complexes had the same shape with lower binding free energy at higher pH, but type II *fA*β–RAGE shifted down approximately 15 kcal/mol (Figure 3). Both complexes were stabilized by lysines 37 and 43 (Figure S2c,d), which are also important for binding DNA [23]. Results indicate that type I fibril had higher affinity for RAGE compared with type II. Type I bound strongly to RAGE independent of pH versus type II, which had lowered affinity as pH decreased.

Binding energy curves for *fA*β-TLR2 complexes showed increased ΔΔG_bind_ with increased pH (Figure 3). For type I interaction, Lys253 was a hot spot independent of pH. Residue Arg32 significantly contributed 2.2 kcal/mol to binding free energy at pH 7 due to two hydrogen bonds formed with His14 and Gln15 of *fA*β (Figure S2e). Within the type II *fA*β–TLR2 complex, residues His202, Lys252, and Lys339 of TLR2 and Asp23 of *fA*β were hot spots independent of pH (Figure S2f). These results indicated both fibrils bound strongly with TLR2 independent of pH.

Binding energy curves for *fA*β–TLR4 complexes showed increased free energy of binding at higher pH. However, the type II curve was shifted down approximately 30 kcal/mol (Figure 3). The type I *fA*β-TLR4 complex was stable at pH 7 with hot spot residues His159, His179, Asp405 of TLR4 and Lys16 and Asp23 of *fA*β (Figure S2g). The type II *fA*β–TLR4 complex did not have any hot spot residues (Figure S2h). These results indicated that type I fibril binds TLR4 under normal conditions, but loses affinity as the environment becomes more acidic.

The above results are compiled in Table S1. In summary, the results highlighted the adaptability of immune receptors at recognizing damaging molecules. TLR4 showed selectivity for the type I structure, while co-receptor CD14 showed selectivity for type II. TLR2 interacted strongly with both fibrils. RAGE interacted more strongly with the type I fibril. Notably, *fA*β–TLR2 complexes and type I *fA*β–RAGE were stable independent of pH. Type I *fA*β–TLR4, type II *fA*β–CD14, and type II *fA*β–RAGE complexes were destabilized as pH decreased.

### 2.2. FAβ Mutants

#### 2.2.1. Are Mutants of Type I or II Structures Feasible?

To assess whether it seems feasible for peptides with common familial mutations to exist as aggregates of type I or II structures, we generated Ala21Gly, Glu22Gly, Glu22Gln, Glu22Lys, and Asp23Asn mutants of type I and II fibrils in silico for a total of 10 mutant fibrils. We calculated mutation energies on stability (ΔΔG_mut,stability_) to compare effects of mutations on the stability of static structures. We performed three 200 ns MD simulations for each mutant fibril and selected representatives for discussion. We limited our analyses to changes in non-bonded interactions indicative of structural integrity, with emphasis on residues of the binding epitope.

**Figure 4:**
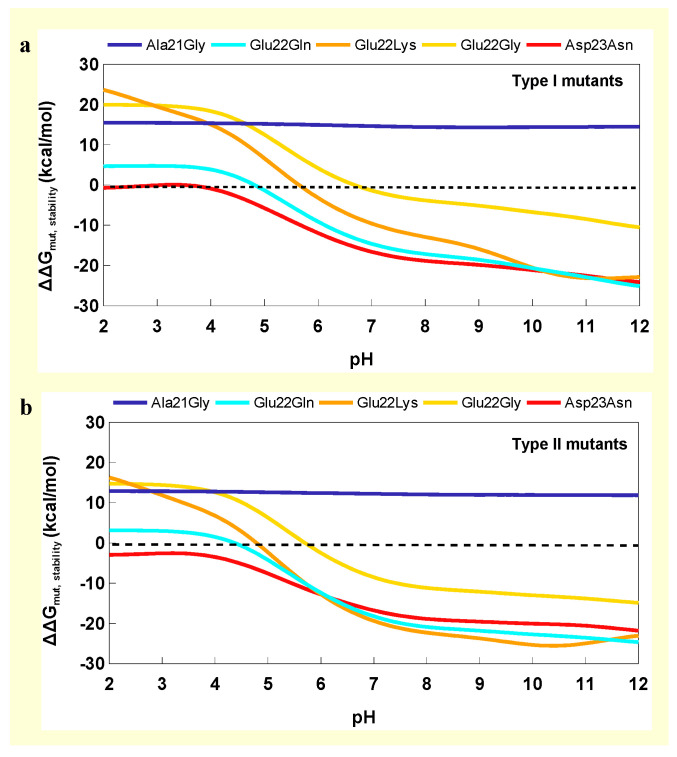
Changes in free energies of mutants as a function of pH for (**a**) type I and (**b**) type II fibril structures. Positive ΔΔG_mut,stability_ indicates destabilizing effects of mutations and negative values indicate stabilizing effects.

The Ala21Gly mutations were highly destabilizing between 12 and 15 kcal/mol, and the effects were independent of pH. Glu22Gly mutation did not affect stability at pH 7 for type I versus type II, which was stabilized by 7 kcal/mol. At pH 5, Glu22Gly fibrils were destabilized by 8 kcal/mol or greater. Both curves had steep slopes from pH 5 to 7, which indicated destabilization of fibrils as pH decreased. Glu22Gln and Asp23Asn mutations were stabilizing by up to 15 or 18 kcal/mol, with increased stability as pH increased. Lysine substitutions were highly stabilizing at higher pH by approximately 8 or 20 kcal/mol for type I and II mutants, respectively. Type I Glu22Lys mutant was destabilized by approximately 6 kcal/mol at pH 5 (Figure 4a,b).

Except for Ala21Gly mutants, mutations were stabilizing at neutral pH. Therefore, MD simulations were carried out at pH 7.4, which allowed for direct comparison with wild-type structures. Interestingly, the results showed all hydrogen bonds that were formed by Ala21 main chain atoms were lost within Gly21 mutants, which further supported the destabilizing effects of the Flemish mutation and suggested that Ala21Gly mutants of type I and II are less feasible. The Glu22Gly mutants were stable throughout two of the MD runs, but each structural mutant seemed less stable during one simulation. The intramolecular non-bonded interactions were similar to wild-type structures, so it was not clear why stability was inconsistent. Therefore, we were unable to assess whether the Gly22 mutation would be feasible for type I or II structures. The remaining Glu22Gln, Glu22Lys, and Asp23Asn mutants of type I and II were stable consistently and are therefore feasible.

#### 2.2.2. What Are the Effects of Familial Mutations on *FA*β Structures?

**Figure 5:**
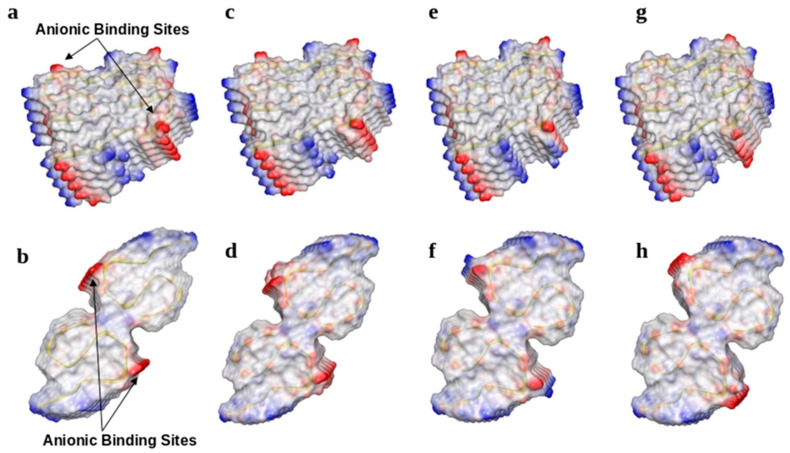
Surface charge maps of type I and type II fibrils with (**a**,**b**) Glu22Gly, (**c**,**d**) Glu22Gln, (**e**,**f**) Glu22Lys, and (**g**,**h**) Asp23Asn mutations. Red indicates negative charge, blue indicates positive charge, and white represents neutral amino acid residues.

To explore the effects of familial mutations on *fA*β structures that were feasible, we employed electrostatic maps to observe differences in surface charge distributions. We assessed the number of contacts with Na^+^ ions, which are indicative of solubility, and in turn we were able to compare with wild-type structures. From surface maps, the Glu22Gly, Glu22Gln, and Asp23Asn mutations decreased the negative surface charge by half (Figure 5a–f). The Glu22Lys mutations caused the largest effects on surface charges due to introduction of positively charged side chains, which enabled the formation of 9 or 11 salt bridges between Lys22 and Asp23 for type I and II mutants, respectively (Figure 5g,h). Notably, these results indicate that mutations affected the charge of the binding epitope and seem to further promote insolubility.

**Figure 6:**
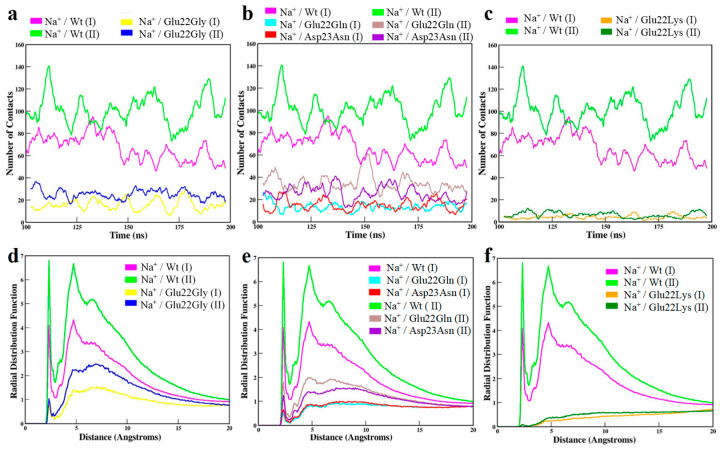
The number of contacts made between side chains of binding epitopes and Na^+^ ions of the solvent for (**a**) Gly22, (**b**) Gln22 and Asn23, and (**c**) Lys22 mutants compared with wild-type fibrils. Contacts are shown as averages calculated every 10 ns. Radial distributions of Na^+^ ions as a function of the distance from residues of the binding epitope for (**d**) Gly22, (**e**) Gln22 and Asn23, and (**f**) Lys22 mutants compared with wild-type fibrils.

We expected mutant fibrils to form half the number of contacts with Na^+^ ions compared to wild-type *fA*β and that radial profiles would be shaped similarly but would have lower peak heights. Instead, Glu22Gly, Glu22Gln, and Asp23Asn mutants formed approximately one-third and Glu22Lys mutants formed about one-tenth the number of contacts with Na^+^ ions compared with wild-types (Figure 6a–c). Radial profiles of glycine, glutamine, and asparagine mutants were shaped as we expected. Curves had a singular distinct peak at 2.5 Å with height 1 or lower, followed by a slight increase to no more than 1.4 at 5 Å. These curves suggest transient interactions that are weak and non-specific (Figure 6d,e). Curves of lysine mutants had no peaks and steadily increased to height value 1 from 0 Å to 20 Å, which indicated binding site residues were not interacting with Na^+^ ions (Figure 6f). This was explained by salt bridges that formed intramolecularly between Lys22 and Asp23 side chains that prevented interactions with solvent molecules. Taken together, these results indicate that the reduced negative charge of the anionic binding further contributes to insolubility of the mutant structures.

In summary, mutations affected electrostatics by decreasing charge strength of the binding epitope and lowering the number of interactions with solvent cations. Because cell surface receptors recognize the negative surface charge of *A*β fibrils, which was affected by mutations, we wanted to study the effects of Glu22Gly, Glu22Gln, Glu22Lys, and Asp23Asn mutations on binding affinity.

#### 2.2.3. Could Mutations Affect Binding Interactions with Proteins?

**Figure 7:**
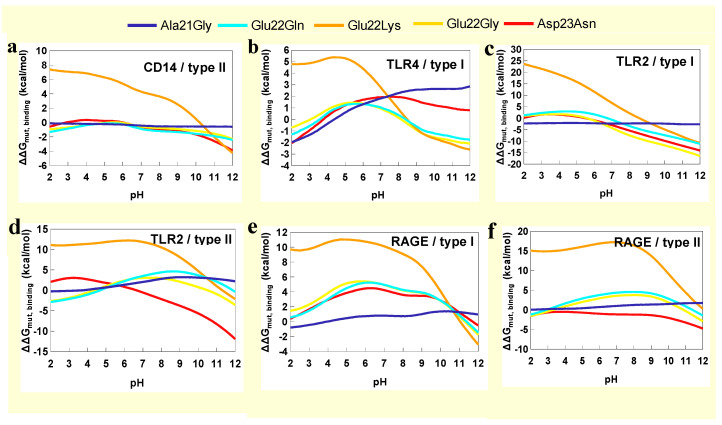
Changes in binding free energies as a response to mutations of (**a**) type II *fA*β–CD14, (**b**) type I *fA*β–TLR4, (**c**) type I *fA*β–TLR2, (**d**) type II *fA*β–TLR2, (**e**) type I *fA*β–RAGE, and (**f**) type II *fA*β–RAGE complexes as a function of pH.

To understand the effects of familial mutations on binding proteins, we performed combinatorial amino acid scanning mutagenesis on *fA*β residues within bound complexes. Here, ΔΔG_mut,binding_ represents the net effect of Glu22Gln, Glu22Lys, Glu22Gly, or Asp23Asn mutations on binding affinity (Figure 7). We focused our analyses on mutations that affected interfacial residues. Mutations that caused ΔΔG_mut,binding_ < −1 kcal/mol were considered stabilizing, and ΔΔG_mut,binding_ > 1 kcal/mol were destabilizing. In an effort to simplify the Discussion section, and taking into account the potential overestimation of destabilization in response to changing multiple strong positive charges to neutral, an arbitrary cutoff of ΔΔG_mut,binding_ ≥ 10 kcal/mol was selected for unstable complexes [30].

The changes in relative binding free energies as a response to mutations (ΔΔG_mut,binding_) quantifies energetic effects primarily to the local environment near the mutated residues. The total binding free energies of *fA*β–protein complexes obtained from MM/GBSA calculations (ΔG_bind_) estimate the binding affinity of fibrils for the receptor with decomposed energetic terms that account for enthalpic and entropic contributions (Tables S1 and S2).

Mutations did not affect stability of the type II *fA*β–CD14 complex except for the Glu22Lys mutation, which was highly destabilizing (Figure 7a). The type I *fA*β–TLR4 interaction had Glu22 and Asp23 residues buried at the interface forming electrostatic interactions with TLR4 residues. Hence Asp23Asn, Glu22Gly, and Glu22Gln mutations were destabilizing independent of pH (Figure 7b). Glu22Gly and Asp23Asn were stabilizing, which could indicate less electrostatic repulsion or formation of stabilizing interactions with TLR2 residues. For example, glycine 22 mutations allow for electrostatic interactions between Asp23 residues with Lys253 and Lys422, and asparagine mutations enable Glu22 interactions with Arg340 and Arg295 (Figure 7c). In type II *fA*β–TLR2 complex, Ala21 and Glu22 residues were not part of the interface. Asp23 residues were at the interface and formed salt bridges with lysine’s 252 and 339. Salt bridges could be replaced by hydrogen bonds with Asp23Asn, which had destabilizing effects that increased as pH decreased with ΔΔG_mut,binding_ equal to 1.8 kcal/mol at pH 5 (Figure 7d).

Type I *fA*β–RAGE had Glu22 and Asp23 residues forming critical interactions independent of pH. Mutations to either residue were destabilizing (Figure 7e). Type II *fA*β–RAGE was destabilized by mutations that disrupted the salt bridge between hot spot Lys37 and Glu22. Glutamine and glycine mutations increased ΔΔG_mut,binding_ between 2 kcal/mol and 5 kcal/mol; lysine substitutions increased ΔΔG_mut,binding_ by ≥10 kcal/mol. Asn23 mutations had no effect. All mutations destabilized interactions with RAGE independent of pH (Figure 7e,f).

In general, vdW potential energy E_vdw_ and nonpolar solvation terms ΔG_solv,SASA_ were similar between wild-type and mutant complexes, and major differences in total binding free energies were attributed to contributions from electrostatic potential energy E_elec_ and polar solvation terms ΔG_solv,GB_ (Table S2). The vdW energy offered greater contributions for fibril interactions with TLRs compared with RAGE. Interactions with TLRs had favorable enthalpic contributions from E_vdw_, while E_elec_ was positive in certain instances making it enthalpically unfavorable; mutations offered improvements for the most part to the entropic cost from ΔG_solv,GB_. Inversely, interactions with RAGE were thermodynamically favorable with more negative ΔG_solv,GB_ values. More specifically, type I mutants had lower affinity for TLR4 compared to wild-type, except for the Glu22Lys mutant, which had −16 kcal/mol lower binding free energy. This is the result of greater electrostatic repulsion at the interface (E_elec_ = 26 kcal/mol), which can be offset by the positive contribution from the solvation free energy (ΔG_solv,GB_ = −286 kcal/mol). Similarly, all mutant–TLR2 complexes had lower ΔG_bind_ and higher estimated affinity compared to wild-type complexes due to positive contributions from ΔG_solv,GB_. Mutant type II *fA*β–RAGE complexes had slightly higher total free energies than the wild-type except for the Glu22Lys mutant, for which the positive contribution from electrostatic potential E_elec_ = −896 kcal/mol outweighed the negative cost from the higher electrostatic solvation free energy ΔG_solv,GB_ = 775 kcal/mol.

More specifically, type I mutants had lower affinity for TLR4 compared to wild-type, except for the Glu22Lys mutant, which had −16 kcal/mol lower binding free energy. This is the result of greater electrostatic repulsion at the interface (E_elec_ = 26 kcal/mol), which can be offset by the positive contribution from the solvation free energy (ΔG_solv,GB_ = −286 kcal/mol). Similarly, all mutant-TLR2 complexes had lower ΔG_bind_ and higher estimated affinity compared to wild-type complexes due to positive contributions from ΔG_solv,GB_. Mutant type II *fA*β–RAGE complexes had slightly higher total free energies than the wild-type, except for the Glu22Lys mutant for which the positive contribution from electrostatic potential E_elec_ = −896 kcal/mol outweighed the negative cost from the higher electrostatic solvation free energy ΔG_solv,GB_ = 775 kcal/mol.

In summary, relative binding free energies as a response to mutations, ΔΔG_mut,binding_, demonstrated direct effects of mutations on specific amino acid interactions at the interface, and observed trends were explained by directly affecting critical residue interactions or hot spots. The differences between ΔΔG_mut,binding_ and total free energies of binding ΔG_bind_ indicate that binding is not dependent upon a specific backbone structure of either the protein or the fibril, which is in line with what has been reported experimentally for *fA*β-protein interactions [13]. The electrostatic potential and polar contributions to desolvation are most important for affinity of type I and II fibrils binding TLRs and RAGE, and the electrostatic potential and polar contributions to desolvation are most important for affinity of type I and II fibrils binding TLRs and RAGE directly. Our study investigates direct interactions between *fA*β and a central binding partner, but environmental factors could also influence binding interactions like the presence or recruitment of accessory proteins or receptor crowding on the cell surface as examples. Notably, the results are in line with experimental reports that showed fibril interactions with macromolecular targets are not specific to amyloid or protein backbones, but are driven by complementary charges between binding epitopes present on both partners [13,31].

## 3. Materials and Methods

### 3.1. Type I and II FAβ Structures

#### 3.1.1. Structure Preparation

*FA*β structures were extracted from the Protein Data Bank (PDB IDs: 7Q4B and 7Q4M) [5]. Structures were prepared using the Calculate Protein Ionization and Residue pK protocol within BIOVIA Discovery Studio 2023 with the CHARMM forcefield applied (Dassault Systèmes BIOVIA, Discovery Studio Modeling Environment, Release 2023, San Diego, CA, USA: Dassault Systèmes, 2023). The CHARMm HBUILD routine was employed to construct coordinates of hydrogen atoms. Residue pKs were calculated at pH 7.4 using the GBPK program with ionic strength 0.145, intramolecular dielectric constant of 10, and energy cutoff 0.9. Ionization states of titratable residues were calculated using the PHPK program, and COO^−^ groups of C termini were kept charged. Prepared *fA*β structures were used as inputs for MD simulations.

Experimental structures of mAb B10, CD14, TLR2, TLR4, and RAGE were extracted from the Protein Data Bank (PDB IDs: 3LN9, 4GLP, 6NIG, 3FXI, and 4OI7) [13,23,32,33,34]. For TLR2 and 4 and RAGE structures, the homodimers of extracellular domains were retained, and all other chains were deleted. For TLRs, monomers were also retained separately. Structures were first prepared similar to type I and II fibrils as described above. Then, proteins were energy minimized using Adopted Basis Newton–Raphson algorithm with maximum 200 steps and RMS gradient 0.1. Prepared protein structures were used as inputs for MD simulations.

#### 3.1.2. MD Simulations and Analysis

Systems were prepared using the Solution Builder utility within CHARMM-GUI v3.7 [35,36]. The type I structure was placed in a 9.1 nm × 9.1 nm × 9.1 nm rectangular box, and the type II structure was placed in a 10.4 nm × 10.4 nm × 10.4 nm rectangular box. Both systems were solvated using the TIP3P water model. All ionizable residues were in their charged state, as appropriate for pH 7 conditions. Each monomer system was net neutral with neutralizing sodium and chloride ions at a 150 mM concentration. The change in the net charge of the fibrillar protein due to the modification of the protonation state resulted in different proportions of sodium versus chloride ions in each system to ensure the global neutrality of the system, keeping the concentration of NaCl at 150 mM. MD simulations were performed using GROMACS-GPU-2023.3 software with CHARMM36 force field [37]. Systems were minimized using steepest descent with maximum of 5000 steps, an initial step size of 0.01 nm, and tolerance of 1000 kJ mol^−1^ nm^−1^. Positional restraints were defined for atoms of *fA*β using the DPOSRES flags for side chain and backbone atoms. The LINCS constraint was applied to set bond lengths [38]. Equilibration stages maintained positional restraints for fibril atoms and employed the md leap-frog integrator with 0.001 time step for 150 ps. The Verlet cutoff scheme was used to generate pair lists updated every 20 steps. The pair list radii were used to treat Lenard Jones potentials and Particle-Mesh Ewald (PME) was used for electrostatics with cutoff distance set to 1.0 nm and 1.2 nm, respectively. Nose–Hoover extended ensemble was used for temperature coupling at 310 K. Production stages also employed the md leap-frog integrator with 0.002 time step for 200 ns. Parrinello–Rahman was used for isotropic pressure coupling with time constant 5 ps. The SETTLE algorithm was applied for treatment of rigid water molecules [39]. Initial runs were extended an additional 300 ns using the gmx convert-tpr function with the -extend keyword. Minimization, equilibration, and production stages of 200 ns were run in four replicas for each system for a total of 1.3 μs per structure.

GROMACS tools used for analysis included (i) gmx sasa for solvent accessible surfaces; (ii) gmx rmsf with fibril C-alpha, side chain, or all atoms selected; (iii) gmx gyrate with -p flag to calculate radii of gyration about the principle axes of fibrils; (iv) gmx rmsd with fibril C-alpha atoms selected; (v) gmx energy to calculate potential energies of fibrils; and (vi) gmx mindist was used to calculate number of contacts between binding epitope residues and solvent molecules. Secondary structure, hydrogen bonds, salt bridges, and radial distribution functions were calculated using VMD 1.9.3 [40]. To extract different conformations of fibrils, trajectory files were combined using the gmx trjcat tool with -cat flag set. To cluster the 10,000 frames, the gmx cluster was used with backbone atoms for least squares fit and RMSD calculations, applying the linkage method with a cutoff set to 0.1 nm. Clusters represented slight differences in disordered residues of the termini, but there were no conformational differences identified in the ordered regions or the binding epitope. We selected two conformations per fibril for molecular docking experiments, which gave comparable results. Thus, our discussion is focused on one of these.

#### 3.1.3. Complex Predictions and Interface Analysis

Low-energy conformations of type I and II structures were docked with mAb B10, CD14, TLR2, and TLR4 monomers, as well as homodimers of TLR2, TLR4, and RAGE using ZDOCK algorithm. In total, 200 poses were generated per docking, and the binding sites were not defined. ZDOCK uses a pairwise shape complementarity search method and scores predicted complexes with geometric descriptors of the Connolly surface [41]. The top 100 ranked poses were re-ranked using the ZRank scoring function which includes short range van der Waals and electrostatic energy terms plus a long range interaction term for charge side chains [42]. The top 100 poses were clustered into 10 groups. Complexes were energy minimized using the CHARMM force field with Adopted Basis Newton–Raphson algorithm with a maximum of 200 steps and RMS gradients of 0.1. We visually inspected the lowest energy complex and the cluster center from each group to select the best poses.

*FA*β–protein interfaces were analyzed with the Analyze Protein Interface protocol within BIOVIA’s Discovery Studio 2023.SP1. Contact area was used to define the interface with probe radius 0.6 to identify interfacial residues based on change in solvent accessible surface area. Additional parameters included maximum hydrogen bond and salt bridge distances set to 3.4 and 4.0 and a neighbor distance cutoff of 5.0. Interfacial residues were optimized using conjugate gradient with a maximum of 200 steps and an RMS gradient of 0.01. Residues that formed non-bonded interactions at the interface were individually mutated to alanine, and the contribution to binding free energy of the complex (ΔΔG_mut_) was estimated using the Calculate Mutation Energy of Binding protocol. Single mutation sites were used, and pH-dependent electrostatics were employed with pH set to 7.4, an ionic strength of 0.1, and energy cutoff of 0.5. ΔG_bind_ is a measure of affinity defined as the electrostatic contribution to the binding free energy of *fA*β–protein complexes, where ΔG_bind_ = ΔG_complex_ − ΔG_protein_ − ΔG*_f_*_*A*β_. Lower ΔG_bind_ indicates higher binding affinity. The ΔΔG_mut_ represents the energetic contribution of an amino acid residue to the binding free energy of the complex, where ΔΔG_mut_ = ΔΔG_bind_(mutant) − ΔΔG_bind_(wild-type) [30].

### 3.2. FAβ Mutants

#### 3.2.1. Generating Mutant *FA*β Structures

Prepared type I and II structures (Section 2.1.1) were used as inputs to perform combinatorial amino- acid scanning mutagenesis on amino acid residues that corresponded to familial mutations Ala21Gly, Glu22Gly, Glu22Gln, Glu22Lys, or Asp23Asn. Because fibrils were dodecamers, 10 mutations were performed per mutagenesis experiment, and 10 experiments were performed in total. The Calculate Mutation Energy (Stability) protocol was used to evaluate the effects of mutations on structure stability. pH-dependent electrostatics were employed with pH set to 7.4, an ionic strength of 0.1, and energy cutoff of 0.5. Briefly, differences in mutation energies (ΔΔG_mut,stability_) represent the effects on conformational stability of fibrils. ΔΔG_mut,sttability_ was calculated by taking the differences in free energy of the folded and unfolded states of *fA*β for the wild-type and mutated structures to obtain the folding free energies (ΔΔG_folding_ = ΔG_folded_ − ΔG_unfold_). Then, the folding free energy of the wild-type is subtracted from the folding free energy of the mutant, which equaled the difference in mutation energy (ΔΔG_mut,stability_ = ΔΔG_folding_ (mutant) − ΔΔG_folding_ (wild-type)) [30]. Conformations of mutated side chains were refined using the ChiRotor algorithm, which employs systematic searching and energy minimization using the CHARMM force field [43]. Refined mutants were used as input for MD simulations (Section 2.2.2).

#### 3.2.2. MD Simulations and Analysis

MD parameters and methods used for analysis were kept the same as described above for type I and II structures, with the exception of the equilibration stage, which was extended to 300 ps. MD calculations were run in triplicate, for a total of 600 ns per mutant fibril.

#### 3.2.3. Generating Mutant *FA*β–Protein Complexes and Interface Analysis

Initially, generated mutants (Section 2.2.1) were docked as described in Section 3.1.3. We found selected poses were superimposable to results of wild-type fibrils. Therefore, we took predicted complexes and performed combinatorial amino-acid scanning on fibril residues to model the effects of Glu22Gly, Glu22Gln, Glu22Lys, or Asp23Asn mutations on protein interactions. Interfacial residues were energy minimized, properties of the interface were calculated, and mutation energies on binding affinity (ΔΔG_mut,binding_) were calculated as described above. The HawkDock server was employed to calculate the total binding free energies (ΔG_bind_) of wild-type and mutant *fA*β–protein complexes using the VD-MM/GBSA method. ΔG_bind_ is the summation of vdW (E_vdw_), electrostatic potential energy (E_elec_), nonpolar solvation energy based on a surface area calculation (ΔG_solv,SASA_), and polar solvation free energy, which is calculated using the Generalized Born model with variable dielectric constants (ΔG_solv,GB_). Nonpolar atoms are given a dielectric constant of 1, polar are assigned a constant of 2, and charged residues are given a dielectric constant of 4 [44].

## 4. Conclusions

Protofibrils are structurally similar and have been shown to interact with immune receptors that were investigated. We performed docking experiments and computational mutagenesis with protofibrils and observed similar binding modes and hot spot residues. Hence, it is reasonable to assume some similarities between protofibrils and fibrils, particularly in regards to the discussed binding interactions. However, the findings cannot be extrapolated to monomers or oligomers that are structurally distant.

We identified differences in structural energetics and metal ion coordination that indicated differences in stability. The type II structure was lower in energy and had stronger coordination networks made up of Glu22 and Asp23 carboxylic acids of either binding epitope compared with type I fibril. We also addressed whether recognition by cell surface receptors could be selective dependent upon fibril structure or system pH. We elucidated binding patterns of cell surface receptors that recognize *fA*β as damaging. Receptor cations were primarily responsible for interacting with carboxylic acids of *fA*β, especially lysines that formed salt bridges with Asp23 residues. Fibril recognition by CD14 and TLR4 was selective dependent upon fibril structure (i.e., type I versus II). TLR2 interacted strongly with both fibrils. RAGE homodimer interacted with both fibrils, however, binding was strongest with type I *fA*β. We concluded that fibril morphology and selectivity of binding are possible explanations for why experimental studies showed discrepant results.

We identified pH-dependent binding for CD14, TLR4, and RAGE interactions where affinity decreased with lowered pH. In contrast, both *fA*β structures had affinity for TLR2 independent of pH. Taken a step further, our results indicate that fibril–protein interactions may be affected by changes in pH. This suggests that mild acidosis occurring either naturally with age or as AD progresses could affect binding modes of *fA*β with cell surface receptors, thereby promoting neurotoxicity.

Further, we explored how common familial AD-associated mutations affected fibril structures and recognition by cell surface receptors. Neither type I nor II structures were feasible for peptides with the Flemish (Ala21Gly) mutation. However, type I or II fibrils composed of peptides with the Dutch (Glu22Gln), Italian (Glu22Lys), or Iowa (Asp23Asn) mutations could be stable, and it is feasible for those structures to exist. All mutations decreased charge strength of the binding epitope, which affected binding affinity. The Glu22Gln, Glu22Gly, and Asp23Asn mutations had similar effects on binding affinity, and the effects differed dependent upon the receptor. Glu22Lys mutations abrogated binding. We concluded that fibril structure also seems to play a role in recognition, where mutations decreased acidity of the binding epitope, altered the electrostatic surface potential, and affected binding affinities. Because mutations consistently lowered affinity of the studied complexes, we speculate that either the higher affinity binding modes differ from those of the wild-type, the binding or orientation of accessory proteins are different, or perhaps a lowered affinity of *fA*β for certain proteins is a general consequence of the studied mutations.

## Data Availability

All relevant data are within the paper and Supplementary Information Material. Raw data will be made available upon request.

## Supplementary Materials

The following supporting information can be downloaded at: https://www.mdpi.com/article/10.3390/molecules30204116/s1, Figure S1: Structural energetics and dynamics of type I and II *fA*β structures. Results from representative MD simulations of type I and II fibrils showing (a) C-alpha RMSDs, (b) root mean squared fluctuations (RMSF) of amino acid residues, and (c) radii of gyration (Rg) of type I and II fibrils.; Figure S2: Free energies of mutations at pH 5 and 7 for (a) type I *fA*β–CD14, (b) type II *fA*β–CD14, (c) type I *fA*β–RAGE homodimer, (d) type II *fA*β–RAGE homodimer, (e) type I *fA*β–TLR2, (f) type II *fA*β–TLR2, (g) type I *fA*β–TLR4, and (h) type II *fA*β–TLR4; Table S1. Summary of amino acid residues contributing most to the binding free energy of selected binding modes. Included residues contributed to complex stability independent of pH (∆∆G_mut_ ≥ 1 kcal/mol at pH 5–7). Interactions formed by stabilizing residues at the interface are provided in column 3, and interactions can involve side chain and/or main chain atoms. *HS* Hot spot residues (∆∆G_mut_ ≥ 2 kcal/mol at pH 5–7). Table S2. Total binding free energies (∆G_bind_) calculated using the variable dielectric MM/GBSA method of selected wild-type and mutant *fA*β-protein complexes. ∆G_bind_ is the summation of van der Waals and electrostatic potential energies (E_vdw_, E_elec_), and polar and non-polar desolvation energies (∆G_solv, GB_, ∆G_solv, SASA_).

## Abbreviations

The following abbreviations are used in this manuscript:

AD	Alzheimer’s disease
*A*β	Beta-amyloid
*fA*β	*A*β fibrils
RAGE	Receptor for Advanced Glycation End products
TLR	Toll-like receptor
Glu22Gly	Arctic mutation
Glu22Gln	Dutch mutation
Asp23Asn	Iowa mutation
Ala21Gly	Flemish mutation
Glu22Lys	Italian mutation
cryo-EM	Cryo-electron microscopy
MD	Molecular dynamics
RMSD	Root mean squared deviation
RMSF	Root mean squared fluctuation
SASA	Solvent accessible surface area
R_g_	Radius of gyration
