# Supplementary material for: Factors Driving Amyloid Beta Fibril Recognition by Cell Surface Receptors: A Computational Study
Source: Molecules. 2025 Oct 17;30(20):4116. doi: 10.3390/molecules30204116 (PMC12566521; doi:10.3390/molecules30204116)
Supplement: Supplementary file 1 [file molecules-30-04116-s001.zip › molecules-3872050-supplementary.pdf]

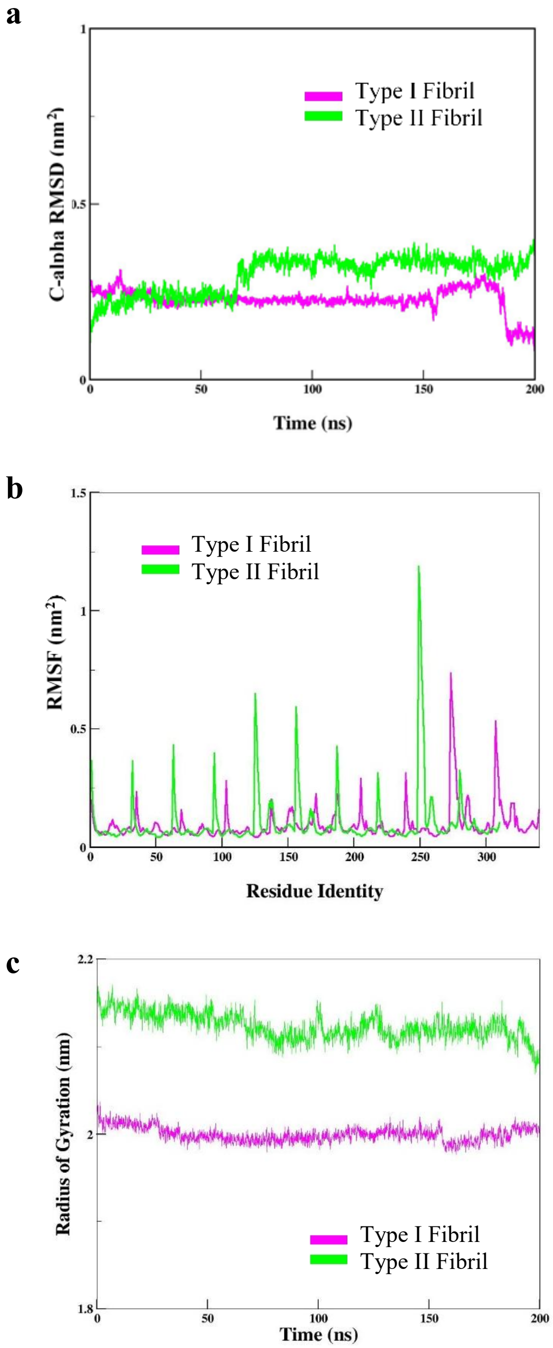

Figure S1: Structural energetics and dynamics of type I and II *f*Aβ structures. Results from representative MD simulations of type I and II fibrils showing (a) C-alpha RMSD's, (b) root mean squared fluctuations (RMSF) of amino acid residues, and (c) radii of gyration (Rg) of type I and II fibrils.

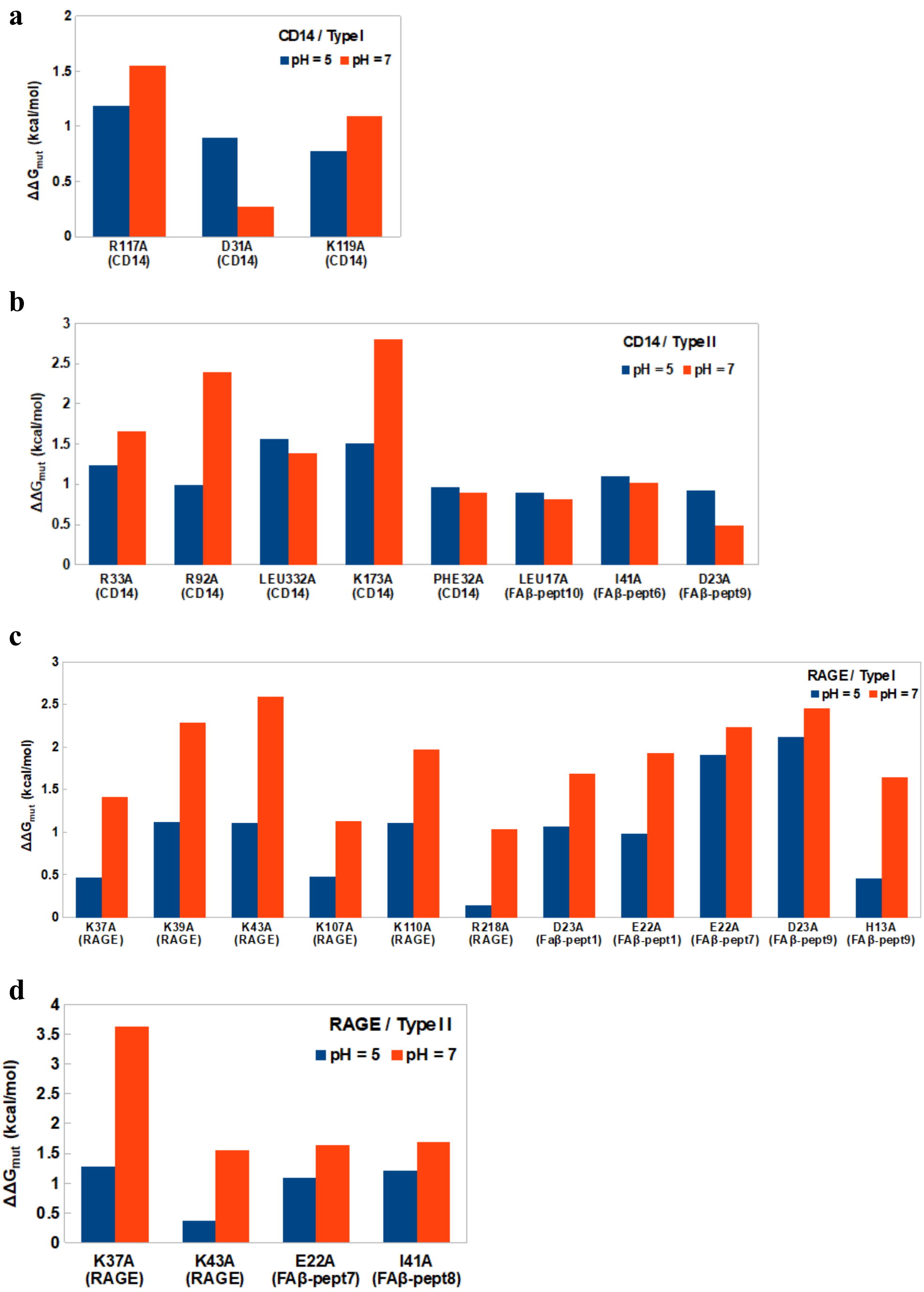

a
CD14 / Type I
pH = 5
pH = 7
ΔΔG_mut (kcal/mol)
0
0.5
1
1.5
2
R117A (CD14)
D31A (CD14)
K119A (CD14)
b
CD14 / Type II
pH = 5
pH = 7
ΔΔG_mut (kcal/mol)
0
0.5
1
1.5
2
2.5
3
R33A (CD14)
R92A (CD14)
LEU332A (CD14)
K173A (CD14)
PHE32A (CD14)
LEU17A (FAβ-pept10)
I41A (FAβ-pept6)
D23A (FAβ-pept9)
c
RAGE / Type I
pH = 5
pH = 7
ΔΔG_mut (kcal/mol)
0
0.5
1
1.5
2
2.5
3
K37A (RAGE)
K39A (RAGE)
K43A (RAGE)
K107A (RAGE)
K110A (RAGE)
R218A (RAGE)
D23A (Faβ-pept1)
E22A (FAβ-pept1)
E22A (FAβ-pept7)
D23A (FAβ-pept9)
H13A (FAβ-pept9)
d
RAGE / Type II
pH = 5
pH = 7
ΔΔG_mut (kcal/mol)
0
0.5
1
1.5
2
2.5
3
3.5
4
K37A (RAGE)
K43A (RAGE)
E22A (FAβ-pept7)
I41A (FAβ-pept8)

e

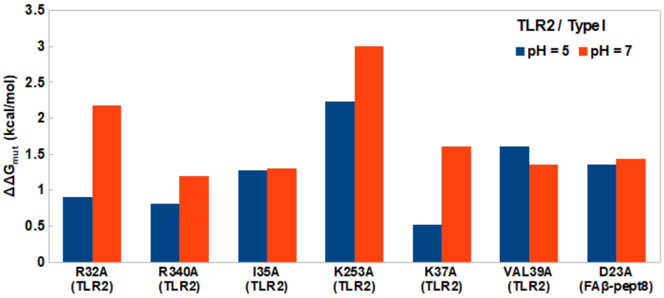

TLR2 / Type I
pH = 5
pH = 7
ΔΔG_mut (kcal/mol)
0
0.5
1
1.5
2
2.5
3
3.5
R32A (TLR2)
R340A (TLR2)
I35A (TLR2)
K253A (TLR2)
K37A (TLR2)
VAL39A (TLR2)
D23A (FAβ-pept8)

f

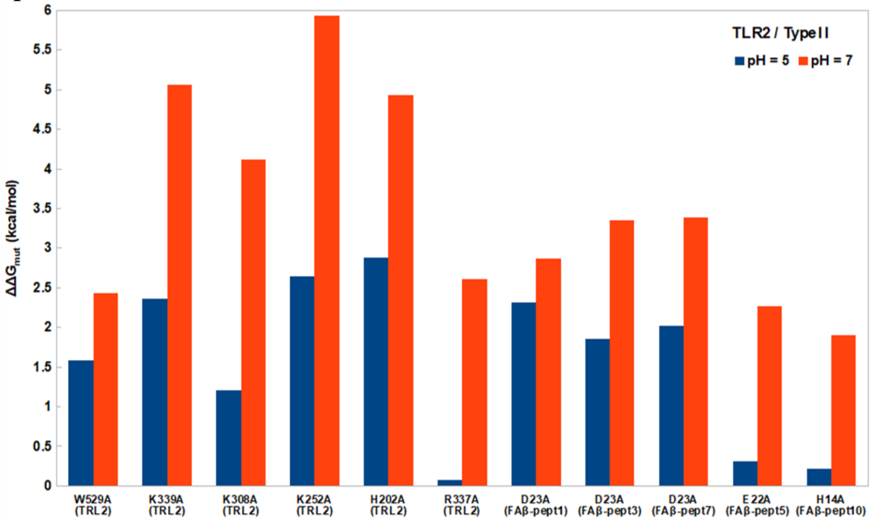

TLR2 / Type II
pH = 5
pH = 7
ΔΔG_mut (kcal/mol)
0
0.5
1
1.5
2
2.5
3
3.5
4
4.5
5
5.5
6
W529A (TRL2)
K339A (TRL2)
K308A (TRL2)
K252A (TRL2)
H202A (TRL2)
R337A (TRL2)
D23A (FAβ-pept1)
D23A (FAβ-pept3)
D23A (FAβ-pept7)
E22A (FAβ-pept5)
H14A (FAβ-pept10)

g

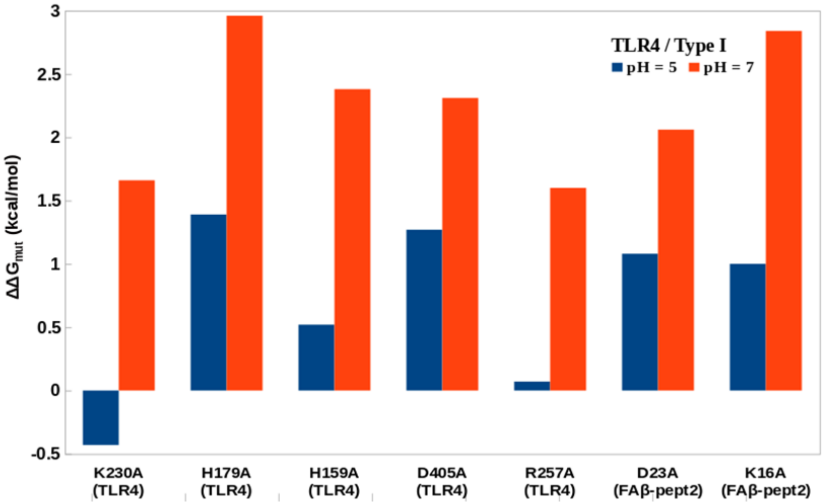

TLR4 / Type I
pH = 5
pH = 7
ΔΔG_mut (kcal/mol)
-0.5
0
0.5
1
1.5
2
2.5
3
K230A (TLR4)
H179A (TLR4)
H159A (TLR4)
D405A (TLR4)
R257A (TLR4)
D23A (FAβ-pept2)
K16A (FAβ-pept2)

h

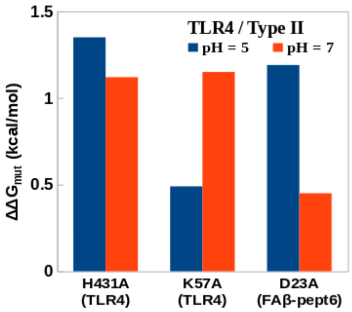

TLR4 / Type II
pH = 5
pH = 7
ΔΔG_mut (kcal/mol)
0
0.5
1
1.5
H431A (TLR4)
K57A (TLR4)
D23A (FAβ-pept6)

Figure S2: Free energies of mutations at pH 5 and 7 for (a) type I *f*Aβ-CD14, (b) type II *f*Aβ-CD14, (c) type I *f*Aβ-RAGE homodimer, (d) type II *f*Aβ-RAGE homodimer, (e) type I *f*Aβ-TLR2, (f) type II *f*Aβ-TLR2, (g) type I *f*Aβ-TLR4, and (h) type II *f*Aβ-TLR4.

Table S1. Summary of amino acid residues contributing most to the binding free energy of selected binding modes. Included residues contributed to complex stability independent of pH ($\Delta\Delta G_{mut}$ ≥ 1 kcal / mol at pH 5-7) . Interactions formed by stabilizing residues at the interface are provided in column 3, and interactions can involve side-chain and/or main-chain atoms.
[HS] Hot spot residues ($\Delta\Delta G_{mut}$ ≥ 2 kcal / mol at pH 5-7).

Complex	Stabilizing Residues of Protein	Stabilizing Residues of Fibril	Interactions Formed by Stabilizing Residues (Protein Residue – Fibril Residue)
**Type I fAβ-CD14**	Arg117	None	Arg117 – Asp23 (peptide 3)
**Type II fAβ-CD14**	Arg33, Arg92, Lys173, Leu332	Ile41 (peptide 6)	Arg33 – Ile41 (peptide 6); Arg92 – Glu22 (peptide 3 and 4); Lys173 – Asp23 (peptide 7);
**Type I fAβ-RAGE**	Lys39, Lys43, Lys110	Glu22 and Asp23 (peptide 1), Glu22 (peptide 7)[HS], Asp23 (peptide 9)[HS]	Lys39 – Glu22 (peptide 1); Lys43 – Glu22 (peptide 7); Asn103 – Glu22 (peptide 7); Lys110 – Asp23 (peptide 1); Lys123 – Asp23 (peptide 9)
**Type II fAβ-RAGE**	Lys37	Glu22 (peptide 7), Ile41 (peptide 8)	Lys37 – Glu22 (peptide 7); Asn25 – Ile41 (peptide 8)
**Type I fAβ-TLR2**	Ile35, Lys253[HS]	Asp23 (peptide 8)	Ile35 – Val39 (peptides 1 and 4); Lys253 – Asp23 (peptide 8)
**Type II fAβ-TLR2**	His202[HS], Lys252[HS], Lys308, Lys339[HS], Trp529	Asp23 (peptide 1)[HS], Asp23 (peptides 3 and 7)	His202 – Ile41 (peptide 6); His202 – Val39 (peptide 6); Lys252 – Asp23 (peptide 1); Lys308 – Glu22 (peptide 1); Lys339 – Asp23 (peptide 7); Trp529 – Leu17 (peptide 10); Trp529 – Val18 (peptide 10)
**Type I fAβ-TLR4**	His179, Asp405	Asp23 (peptide 2)	His179 – Glu22 (peptide 8); Asp405 – Asn27 (peptide 4); Arg289 – Asp23 (peptide 2)
**Type II fAβ-TLR4**	His431	None	His431 – Asp23 (peptide 1)

Table S2. Total binding free energies (ΔG_{bind}) calculated using the variable dielectric MM/GBSA method of selected wild-type and mutant *f*Aβ-protein complexes. ΔG_{bind} Is the summation of van der Waals and electrostatic potential energies (E_{vdw}, E_{elec}), and polar and non-polar desolvation energies ($\Delta G_{solv, GB}$, $\Delta G_{solv, SASA}$).

Complex	Mutation	E_{vdw} (kcal / mol)	E_{elec} (kcal / mol)	$\Delta G_{solv, GB}$ (kcal / mol)	$\Delta G_{solv, SASA}$ (kcal / mol)	ΔG_{bind} (kcal / mol)
Type I *f*Aβ-TLR4	Wild-type	-263	-1,236	993	-33	-539
	Ala21Gly	-262	-1,729	1,515	-33	-509
	Glu22Gly	-239	-1,022	807	-29	-483
	Glu22Gln	-266	-947	722	-32	-524
	Glu22Lys	-262	26	-286	-33	-555
	Asp23Asn	-263	-934	707	-32	-521
Type I *f*Aβ-TLR2	Wild-type	-218	636	-801	-30	-413
	Ala21Gly	-218	428	-858	-31	-679
	Glu22Gly	-195	487	-890	-27	-624
	Glu22Gln	-220	514	-963	-31	-700
	Glu22Lys	-217	702	-1,250	-32	-797
	Asp23Asn	-211	638	-1,075	-31	-678
Type II *f*Aβ-TLR2	Wild-type	-242	-221	-6	-30	-499
	Ala21Gly	-241	-135	-99	-30	-504
	Glu22Gly	-242	147	-392	-29	-516
	Glu22Gln	-247	137	-379	-30	-518
	Glu22Lys	-247	466	-715	-30	-525
	Asp23Asn	-247	312	-567	-30	-532
Type I *f*Aβ-RAGE	Wild-type	-171	3,262	-3,430	-25	-364
	Ala21Gly	-169	3,566	-3,720	-25	-348
	Glu22Gly	-74	1,216	-1,570	-24	-451
	Glu22Gln	-84	1,210	-1,621	-26	-521
	Glu22Lys	-171	-622	453	-24	-364
	Asp23Asn	-82	1,426	-1,794	-26	-477
Type I *f*Aβ-RAGE	Wild-type	-144	3,711	-3,824	-20	-278
	Ala21Gly	-139	3,650	-3,758	-19	-267
	Glu22Gly	-136	1,312	-1,419	-18	-261
	Glu22Gln	-145	1,368	-1,476	-19	-273
	Glu22Lys	-143	-896	775	-20	-284
	Asp23Asn	-141	1,404	-1,509	-19	-266
